# Supplementary material for: Both microRNA-455-5p and -3p repress hypoxia-inducible factor-2α expression and coordinately regulate cartilage homeostasis
Source: Nat Commun. 2021 Jul 6;12:4148. doi: 10.1038/s41467-021-24460-7 (PMC8260725; doi:10.1038/s41467-021-24460-7)
Supplement: Supplementary file 1 — Supplementary information [file 41467_2021_24460_MOESM1_ESM.pdf]

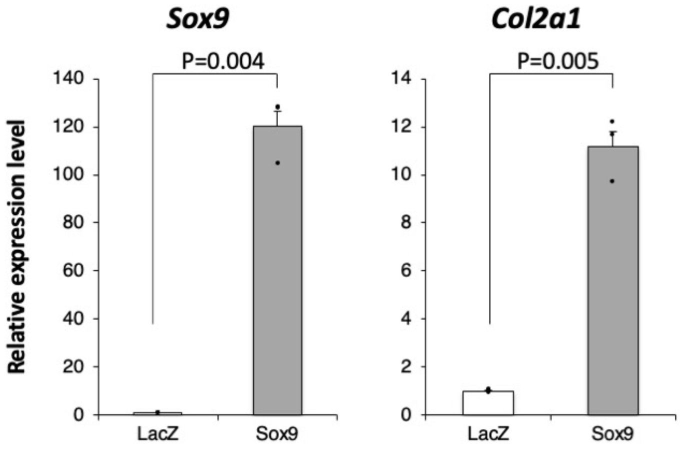

Supplementary Fig. 1. Relative mRNA levels of *Sox9* and *Col2a1* in adenovirus-infected chondrocytes.

Sox9- or LacZ-expressing adenovirus were infected into mouse primary chondrocytes at MOI 50. The experiment was performed once with 3 biological replicates (n=3). Data are represented as the mean ± SEM. Two-tailed Student's t-test.

Source data are provided as a Source Data file.

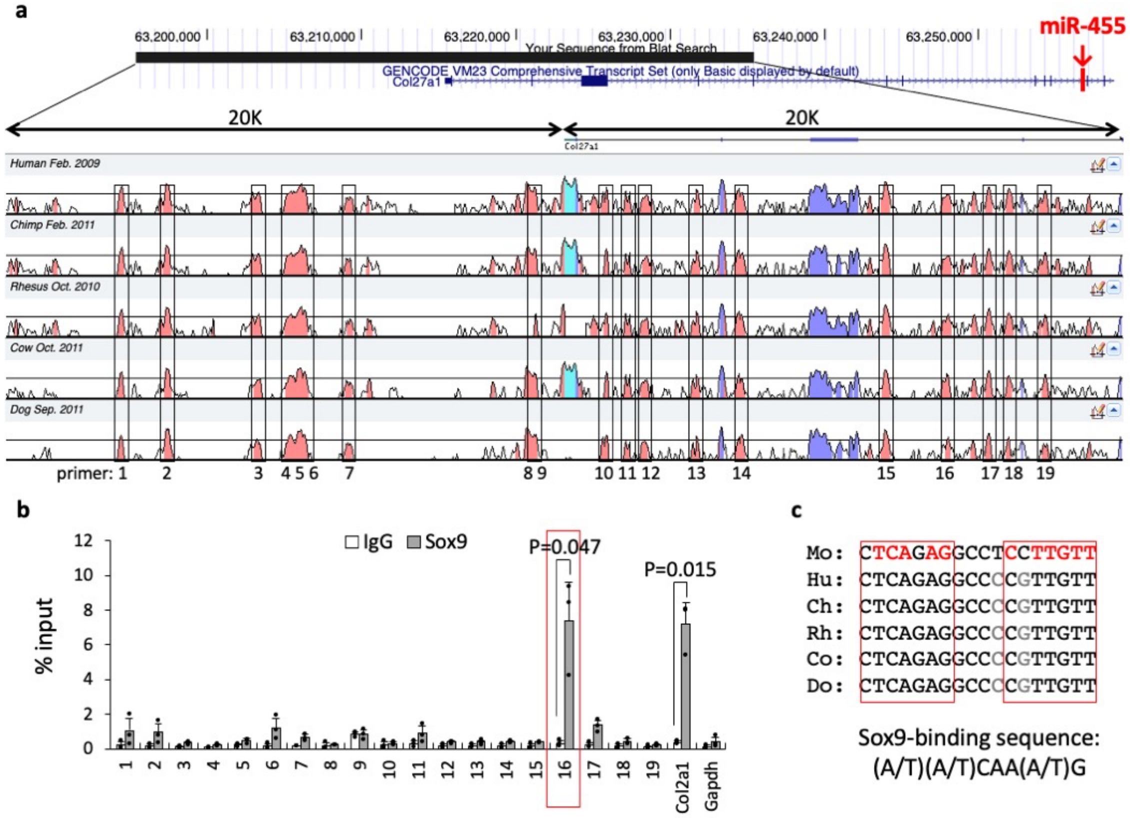

Supplementary Fig. 2. ChIP analysis using anti-Sox9 antibody on the *Col27a1* gene locus in mouse chondrocytes.

(a) Conservation analysis of the mouse *Col27a1* gene locus by VISTA-point, and positions of the primer sets for the ChIP analysis (below).

(b) Quantitative ChIP analysis using anti-Sox9 antibody in mouse chondrocytes. This assay was performed independently three times (n=3). Data are represented as the mean ± SEM. Two-tailed Student's t-test.

(c) Sox9-binding consensus-like sequence of primer set no. 16 region in *Col27a1* intron 3. Mo, mouse; Hu, human; Ch, chimpanzee; Rh Rhesus; Co, cow; Do, dog.

Source data are provided as a Source Data file.

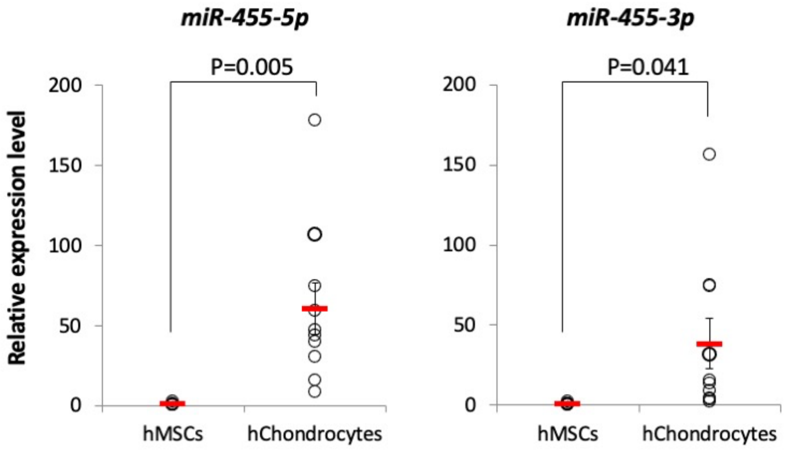

Supplementary Fig.3. Relative mRNA levels of miR-455-5p (455-5p) and -3p (455-3p) in human (h) MSCs (n=7, biologically independent samples) or chondrocytes (n=10, biologically independent samples). Data are represented as the mean ± SEM. Two-tailed Student's t-test.

Source data are provided as a Source Data file.

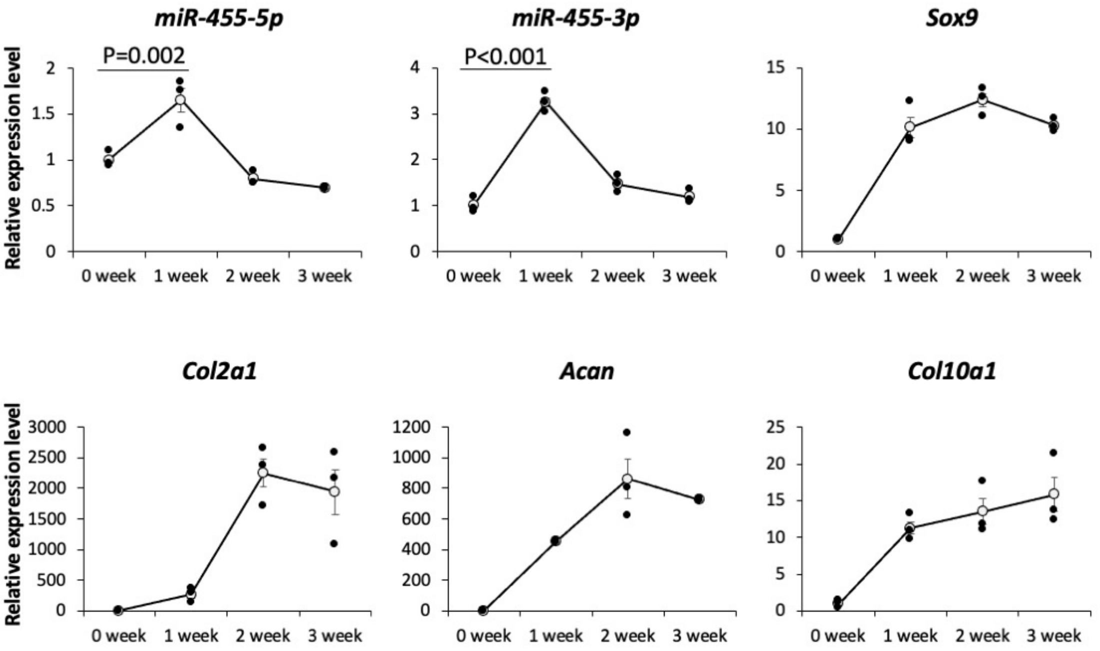

Supplementary Fig. 4. Relative expression levels of miR-455s and cartilage marker genes during mouse MSC chondrogenesis in pellet cultures. The experiment was performed once with 3 biological replicates (n=3). Data are represented as the mean ± SEM. Two-tailed Dunnett's test.

Source data are provided as a Source Data file.

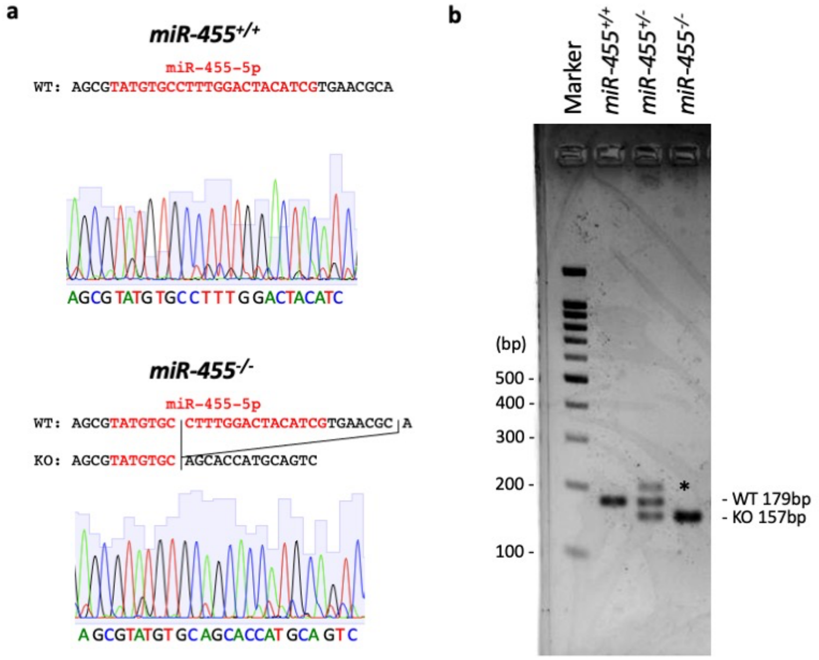

Supplementary Fig. 5. Genotyping of miR-455 knockout mice.

(a) Sequence of miR-455 gene locus in wild-type (WT) or miR-455 knockout (KO) mice.

(b) Genotyping PCR data of miR-455$^{+/+}$, miR-455$^{+/-}$, and miR-455$^{-/-}$ mice. Genotyping PCR data was confirmed by at least 5 miR-455 mutant mice that confirmed the genotype by genome sequencing. * non-specific band.

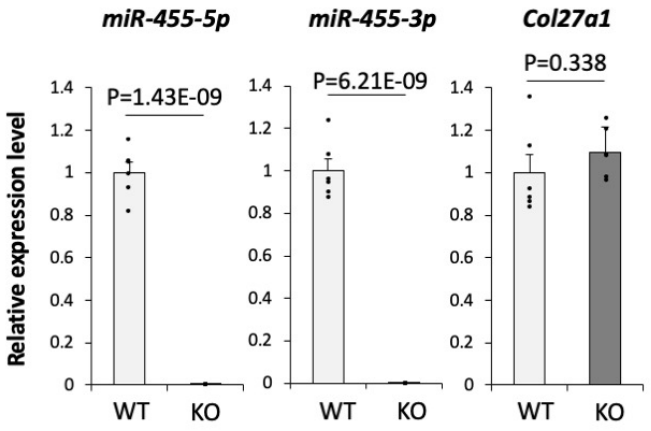

Supplementary Fig. 6. Relative expression levels of miR-455s and *Col27a1* in wild-type (WT) or miR-455 knockout (KO) primary chondrocytes. The experiment was performed once with 3 biological replicates (n=3). Data are represented as the mean ± SEM. Two-tailed Student's t-test.

Source data are provided as a Source Data file.

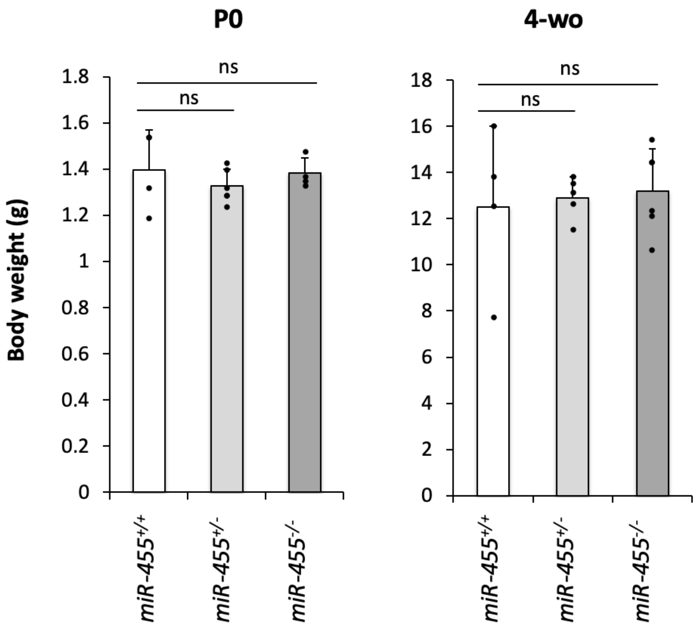

Supplementary Fig. 7. Body weight of post neonatal day 0 (P0) and 4-week-old (4wo) miR-455$^{+/+}$, miR-455$^{+/-}$ or miR-455$^{-/-}$ mice. Error bars show SD. P0 (sex undetermined): miR-455$^{+/+}$, n=4; miR-455$^{+/-}$, n=6; miR-455$^{-/-}$, n=4. 4-wo (female): miR-455$^{+/+}$, n=4; miR-455$^{+/-}$, n=5; miR-455$^{-/-}$, n=6, biologically independent samples. Data are represented as the mean ± SD. ns: not significant. Two-tailed Dunnett's test.
Source data are provided as a Source Data file.

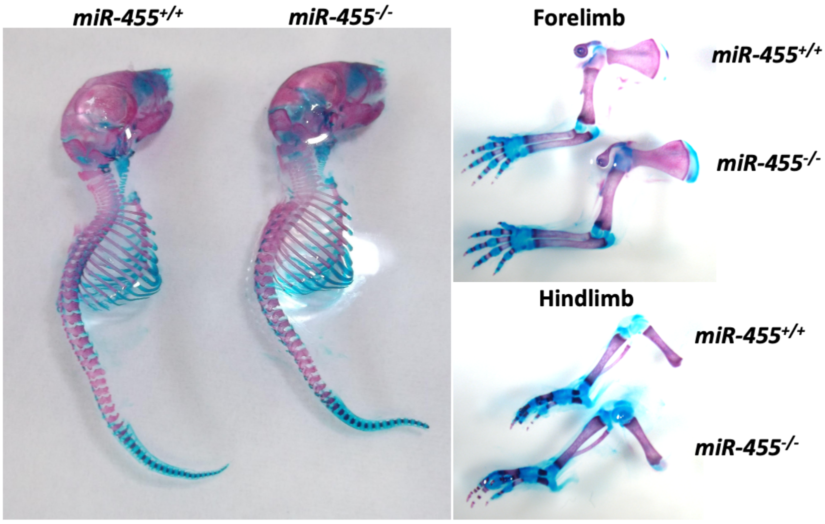

Supplementary Fig. 8. Skeletal prep of miR-455$^{+/+}$ or miR-455$^{-/-}$ mice (post neonatal day 2).

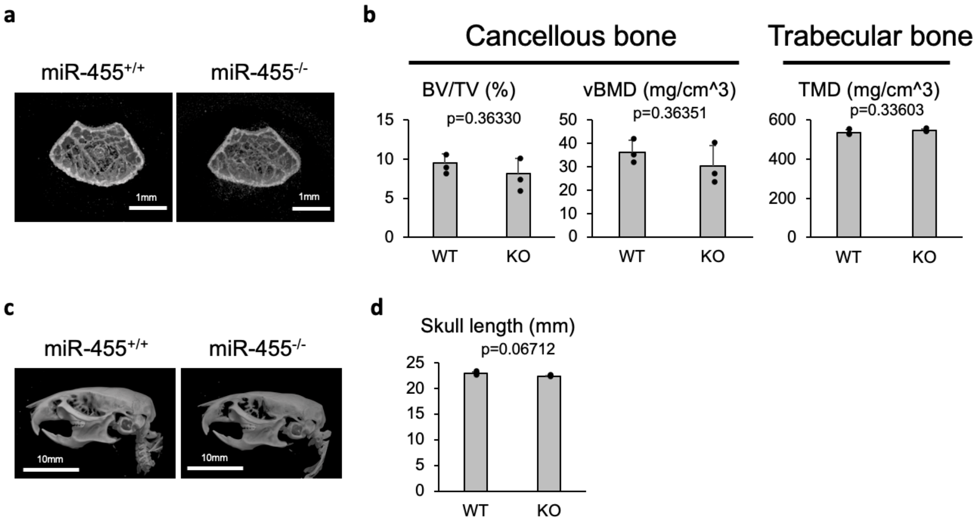

Supplementary Fig. 9. Micro-CT analyses of miR-455$^{+/+}$ or miR-455$^{-/-}$ male mice (8-week-old).

(a) Micro-CT images of femur in miR-455$^{+/+}$ or miR-455$^{-/-}$ mice. The result was similar with three independent samples. scale bars show 1 mm.

(b) Trabecular bone volume fraction (BV/TV) and volumetric BMD (vBMD) of cancellous bone and tissue mineral density (TMD) of trabecular bone in wild-type (WT) and miR-455 knockout (KO) femur. Data are represented as the mean ± SD, n=3, biologically independent samples. Two-tailed Student's t-test.

(c) Micro-CT images of skull in miR-455$^{+/+}$ and miR-455$^{-/-}$ mice. The result was similar with three independent samples. scale bars show 10 mm.

(d) Skull length of wild-type (WT) and miR-455 knockout (KO) mice. Data are represented as the mean ± SD, n=3, biologically independent samples. Two-tailed Student's t-test.

Source data are provided as a Source Data file.

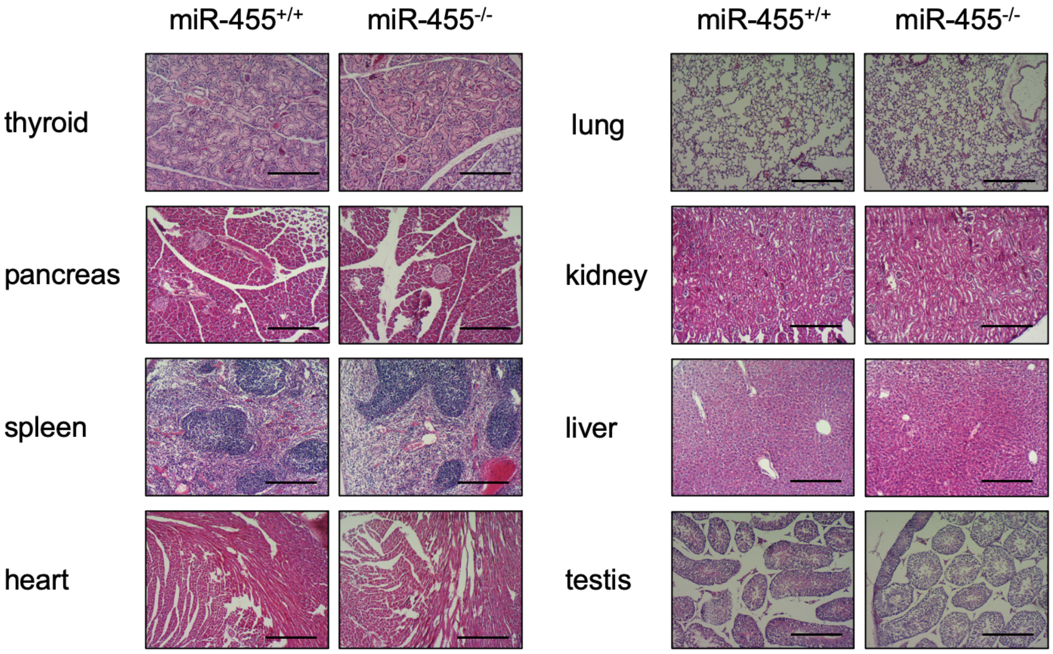

Supplementary Fig. 10. Representative images of H&E staining for major tissues of miR-455$^{+/+}$ or miR-455$^{-/-}$ male mice (8-week-old). The result was similar with three independent samples. Scale bar shows 600 μm.

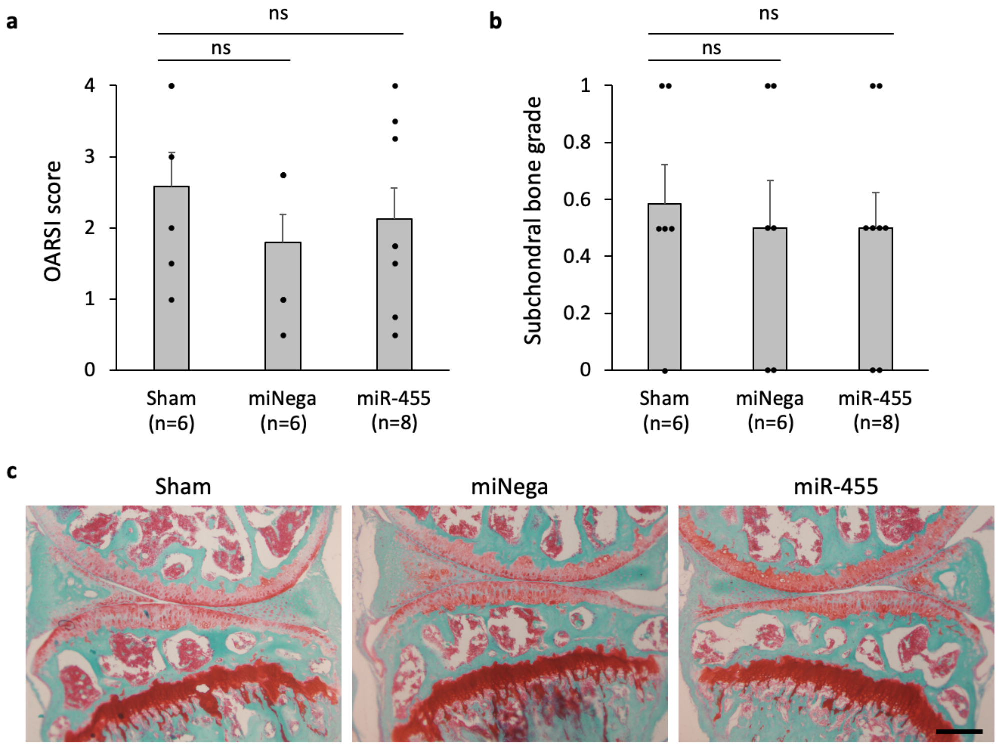

Supplementary Fig. 11. Phenotype analysis of sham or miRNA control mimic (miNega) or miR-455-5p/3p (miR-455) injected-knee joint.

(a) The OARSI scores of sham (n=6, biologically independent samples) or miRNA control mimic (miNega; n=6, biologically independent samples) or miR-455-5p/3p (miR-455; n=8, biologically independent samples) injected knee joints. Data are represented as the mean ± SEM. ns: not significant. One-tailed Dunnett's test.

(b) The subchondral bone scores of sham (n=6, biologically independent samples) or miRNA control mimic (miNega; n=6, biologically independent samples) or miR-455-5p/3p (miR-455; n=8, biologically independent samples) injected knee joints. Data are represented as the mean ± SEM. ns: not significant. One-tailed Dunnett's test.

(c) Representative image of Safranin O staining of miRNA mimic-injected knee joints. Staining was repeated at least twice with similar results. Scale bar shows 200 μm.

Source data are provided as a Source Data file.

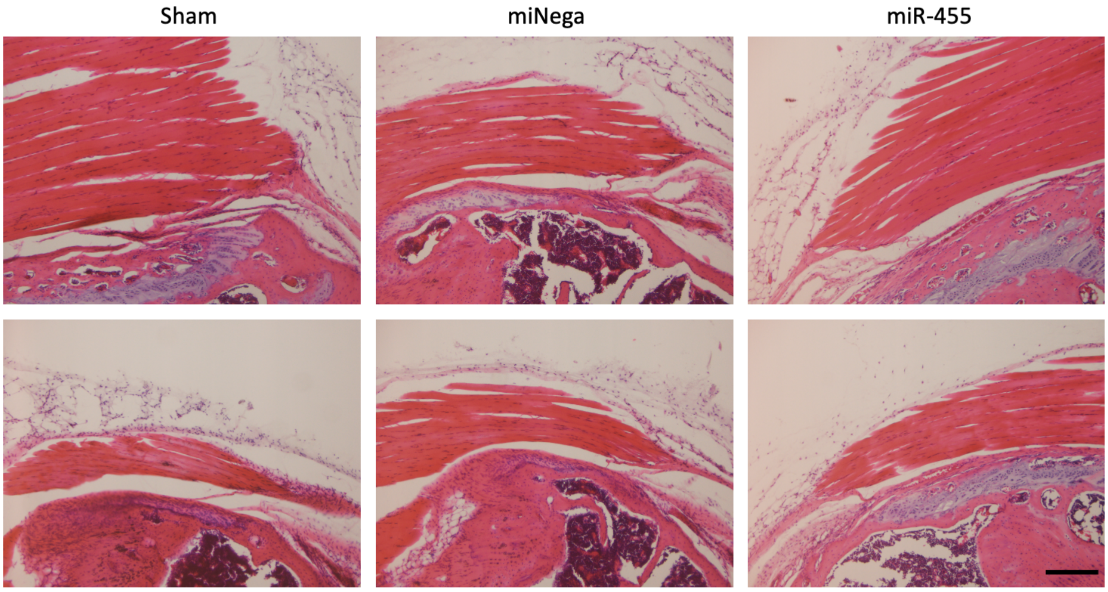

Supplementary Fig. 12. Representative image of H&E staining for quadriceps of sham or miRNA control mimic (miNega) or miR-455-5p/3p (miR-455) injected knee joint. The result was similar with at least three independent samples. Scale bar shows 200 μm.

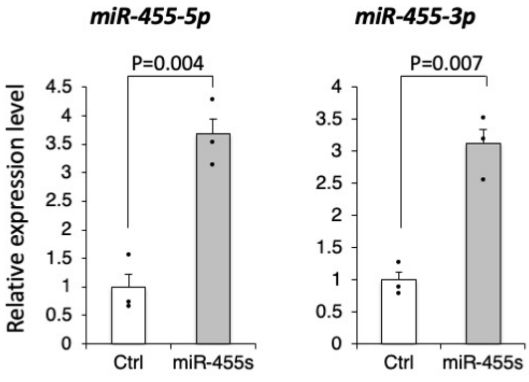

Supplementary Fig. 13. Relative expression of miR-455s in negative control mimic (Ctrl) or miR-455s mimic (miR-455s) transfected articular cartilage of C57BL6 mice. The experiment was performed once with 3 biological replicates (n=3). Data are represented as the mean ± SEM. Two-tailed Student's t-test.

Source data are provided as a Source Data file.

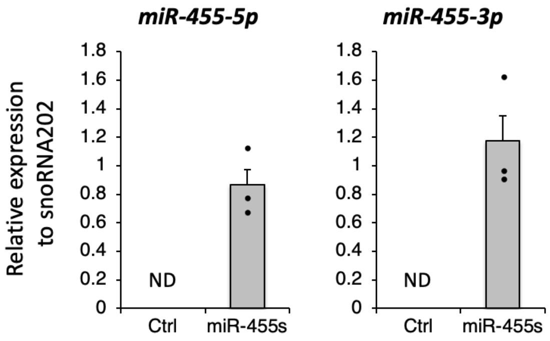

Supplementary Fig. 14. Relative expression of miR-455s (normalized to snoRNA202) in negative control mimic (Ctrl) or miR-455s mimic (miR-455s) transfected articular cartilage of 4-month-old miR-455 knockout mice. The experiment was performed once with 3 biological replicates (n=3). Data are represented as the mean ± SEM. ND: not detected.

Source data are provided as a Source Data file.

Supplementary Table 1. Primer sequences for ChIP

Primer set	Forward	Reverse
Col2a1 Sox9 binding site	CACTGGGCCTTGCCTCTCATG	CGATGGCTTCCAGATGGGCTG
Col27a1 primer no.1	GCAGTGGCAACCTCATTGTTC	CCCAGTGACTTGCAGAAACTAGC
Col27a1 primer no.2	CCCTAGATGGAGCCAGCAAAC	GGTGCAGGTACTTACTGGTGC
Col27a1 primer no.3	CGTGGAAACCTCTTCCTCCAAG	GTGCAAGGTTCTGAGGCCATG
Col27a1 primer no.4	GAGTGACCCAGGGCTTGGAG	GGGCTTCGCTGGCCTATAAAC
Col27a1 primer no.5	CGTGGTGGCAGACAGCTTCC	CACATTCTCCCCATCTCTCAGG
Col27a1 primer no.6	GCGTATGTGGCTGAAACCTCC	CTCTCTGGGCCTCAGTTTCC
Col27a1 primer no.7	CATCCAGGGAGACAGCCCTC	CCAGGACAGAGGGAAGTTTGC
Col27a1 primer no.8	CTCCGACTTGGGACTTTTCCG	GGTGGCTCATAGGTGCTGAG
Col27a1 primer no.9	CTCGAGCTGGAGCCGGAG	CAGAGCTCCGTGAGTCCCAG
Col27a1 primer no.10	GGCCAGAGTTATAGTACCGCC	CTTTGGCACCACCACACTGC
Col27a1 primer no.11	CACAGCTGCTATTTGTATCAGTGC	CTCTGTGGCCTCATCAGACC
Col27a1 primer no.12	AGCCAAGGTCTGTGAAGCTG	GGATTGGAGATGCAACCAGGAG
Col27a1 primer no.13	GAAGAGGAACCTGGCCAGTG	GTGGGAGCAGCCACTGGAAC
Col27a1 primer no.14	CACCTCAGGAGTCCCCATGG	CTGGCTCGGTACAGCTCCGG
Col27a1 primer no.15	GTTTGAGGCTCAGGCACCTTG	GAGCAGACTCTGTCCCCTTG
Col27a1 primer no.16	CCTGCACTCCCTGGACAGAG	CTGCTCCATCTGAGTGAGGC
Col27a1 primer no.17	CCCAGTGTGAGAGCGCGATC	GCAAACGTGCATGCAAGAGAC
Col27a1 primer no.18	GTGCGAGGATGCTTGGTGTG	GAGTCATCCTACCCCTAGGG
Col27a1 primer no.19	CATGACAAGGGCCAGGCAAG	GCAGGTTCTGCAGCCAAGAG
Gapdh	CCTGGTCACCAGGGCTGC	CGCTCCTGGAAGATGGTGATG

Supplementary Table 2. Gross size analysis of various tissues in 8-week-old miR-455 knockout mice

		WT1	WT2	WT3	KO1	KO2	KO3	Average		SD		p value
								WT	KO	WT	KO	
Height	mm	88.27	87.5	88.52	87.96	87.86	88.76	88.1	88.19	0.43	0.4	0.83
Weight	g	27.3	26.7	27.6	29	27.3	30.6	27.2	28.97	0.37	1.35	0.15
Subcutaneous fat	mm	0.4	0.44	0.44	0.49	0.46	0.46	0.43	0.47	0.02	0.01	0.06
Lower limb length	mm	37.54	37.64	39.15	39.54	37.44	37.58	38.11	38.19	0.74	0.96	0.93
Thickness of the quadriceps muscle	mm	3.69	3.82	3.67	3.62	3.72	3.63	3.73	3.66	0.07	0.04	0.29
Tooth length	mm	4.19	4.3	4.16	4.16	3.97	4.01	4.22	4.05	0.06	0.08	0.08
Abdominal girth	mm	68.33	67.02	64.03	69.63	68.97	69.47	66.46	69.36	1.8	0.28	0.09
Thyroid	g	0.17	0.31	0.22	0.26	0.28	0.38	0.23	0.31	0.06	0.05	0.26
Lung	g	0.29	0.34	0.34	0.34	0.36	0.45	0.32	0.38	0.02	0.05	0.19
Heart	g	0.13	0.15	0.13	0.14	0.14	0.15	0.14	0.14	0.01	0	0.42
Liver	g	2.2	2.05	2.28	2.38	1.76	2.4	2.18	2.18	0.1	0.3	0.99
Pancreas	g	0.14	0.23	0.27	0.16	0.2	0.19	0.21	0.18	0.05	0.02	0.5
Spleen	g	0.17	0.16	0.16	0.16	0.18	0.16	0.17	0.17	0	0.01	0.68
Kidney (Rt)	g	0.26	0.29	0.31	0.36	0.27	0.32	0.29	0.32	0.02	0.04	0.37
Testis	g	0.2	0.24	0.16	0.2	0.13	0.11	0.2	0.15	0.03	0.04	0.21

Supplementary Table 3. Primer sequences for RT-qPCR

Gene	Forward	Reverse
Gapdh	CCTGGTCACCAGGGCTGC	CGCTCCTGGAAGATGGTGATG
pri-miR-455	GCTTCCTTCCACAGGTCGCG	CTCTGTGGTGGTGGCTCCAG
Col27a1	CTCCACCCAAGGAGCTCCTG	CCCAGGGTGGTGGGAAGAAC
Sox9	GCAGGAAGCTGGCAGACCAG	GCTGCACGCGCAGCCGCTCC
Col2a1	CAGGTGAACCTGGACGAGAG	ACCACGATCTCCCTTGACTC
Col10a1	GAGATGCATTTGGAGGTAGG	GATTGCTGAGTGCTCCGGAG
Acan	GGTCATCGCTGCAGTGATCTC	GCAGTGGTCACAGGATGCATG
Mmp3	GCCTATGCACCTGGACCAGG	CTGTGGAGGACTTGTAGACTGGG
Mmp13	CAGTTGACAGGCTCCGAGAA	TTCACCCACATCAGGCACTC
Adamts5	CCAGCATCGATGCATCCAAGC	CTGTCCTGGGAGTTCCTCGG
Nos2	TCACCACAAGGCCACATCGG	CAATGGCATGAGGCAGGAGC
Epas1	CACGGCGACAATGACAGCTG	GGGGCAACTCATGAGCCAAC
Aph1a	CACTGTAGCTGGAGACCCAC	CCGTGCATCTGATCGGTCTG
Fbxl2	CAACTGGCAACGGGTGGATC	CGGCAGTTCTGTGCAAAGGTC
Fkbpl	CTGTGACTTAGGCCGTCCTG	CGCTGCCTAAACGGAATGGC
Zbtb20	CCTCATCTGCAGGCCACAGC	CGCCAGCAGATGCTGCACTG

Supplementary Table 4. Primer sequences for reporter constructions.

pLuc2 3U universal R	GAATTATTACACGGCGATCTTGCCG
EPAS1 3U F	GCCAGGCCTTCTACCTGGGC
APH1A 3U F	CTACCTGGACTGATCGCCTG
FBXL2 3U F	TGACAGCAGCTGCCTGGG
ZBTB20 3U F	GTGTCTGACGGATAAGTAGTATCTTTCTC
APH1A CDS F	TTTGAATTCATGGGGGCTGCGGTGTTTTTC
APH1A CDS R	TTTACTAGTCAGTCCTTACACAAGAGGCTGC
FBXL2 CDS F	AAATCTAGAGTTTTCTCAAACAATGATGAAGGCC
FBXL2 CDS R	AAAGAATTCAGAGAATGACACAGCACCTG
FKBPL CDS F	TTTGAATTCATGGAGACGCCACCAGTCAATAC
FKBPL CDS R	TTTACTAGTCAGCCAAACATCTTGCGCAG
ZBTB20 CDS F	AAATCTAGATGACCGAGCGCATTCACAG
ZBTB20 CDS R	AAAGAATTCTTATCCGTCAGACACATGCATCC
EPAS1 3U 5p mut F	GCGTCATTTGGACATTTCCAGAACTAC
EPAS1 3U 5p mut R	GTGTGAAAGACCATCCGAGTCAC
EPAS1 3U 3p mut F	CTGCTGTGAGGAGGAGGCTGCAC
EPAS1 3U 3p mut R	CTCCACGGGGCTCCAAGGTC
