# Supplementary material for: Both microRNA-455-5p and -3p repress hypoxia-inducible factor-2α expression and coordinately regulate cartilage homeostasis
Source: Nat Commun. 2021 Jul 6;12:4148. doi: 10.1038/s41467-021-24460-7 (PMC8260725; doi:10.1038/s41467-021-24460-7)
Supplement: Supplementary file 2 — Description of Additional Supplementary Files [file 41467_2021_24460_MOESM2_ESM.pdf]

Title: Supplementary Data 1.

Description: Microarray data on all miRNAs whose signals are detected by lacZ- or Sox9- expressing adenovirus infection in chondrocytes. LacZ- or Sox9-expressing adenovirus were infected in chondrocytes at MOI 5 or 20. Mature miRNAs were selectively labeled and hybridized on Mouse miRNA Microarray Release 15.0 (Agilent Technology) using microRNA Labeling and Hybridization kit (Agilent Technology).
