# Supplementary material for: Both microRNA-455-5p and -3p repress hypoxia-inducible factor-2α expression and coordinately regulate cartilage homeostasis
Source: Nat Commun. 2021 Jul 6;12:4148. doi: 10.1038/s41467-021-24460-7 (PMC8260725; doi:10.1038/s41467-021-24460-7)
Supplement: Supplementary file 3 — Reporting Summary [file 41467_2021_24460_MOESM3_ESM.pdf]

nature research

Corresponding author(s): Hiroshi Asahara

Last updated by author(s): Mar 11, 2021

Reporting Summary

Nature Research wishes to improve the reproducibility of the work that we publish. This form provides structure for consistency and transparency in reporting. For further information on Nature Research policies, see our Editorial Policies and the Editorial Policy Checklist.

Statistics

For all statistical analyses, confirm that the following items are present in the figure legend, table legend, main text, or Methods section.

n/a	Confirmed	
☐	☒	The exact sample size (*n*) for each experimental group/condition, given as a discrete number and unit of measurement
☐	☒	A statement on whether measurements were taken from distinct samples or whether the same sample was measured repeatedly
☐	☒	The statistical test(s) used AND whether they are one- or two-sided<br>*Only common tests should be described solely by name; describe more complex techniques in the Methods section.*
☒	☐	A description of all covariates tested
☐	☒	A description of any assumptions or corrections, such as tests of normality and adjustment for multiple comparisons
☐	☒	A full description of the statistical parameters including central tendency (e.g. means) or other basic estimates (e.g. regression coefficient) AND variation (e.g. standard deviation) or associated estimates of uncertainty (e.g. confidence intervals)
☐	☒	For null hypothesis testing, the test statistic (e.g. *F*, *t*, *r*) with confidence intervals, effect sizes, degrees of freedom and *P* value noted<br>*Give P values as exact values whenever suitable.*
☒	☐	For Bayesian analysis, information on the choice of priors and Markov chain Monte Carlo settings
☒	☐	For hierarchical and complex designs, identification of the appropriate level for tests and full reporting of outcomes
☒	☐	Estimates of effect sizes (e.g. Cohen's *d*, Pearson's *r*), indicating how they were calculated

Our web collection on statistics for biologists contains articles on many of the points above.

Software and code

Policy information about availability of computer code

Data collection	No software was used for data collection.
Data analysis	Dunnett's tests and Tukey-Kramer tests were performed using RStudio.Version (3.5.1).

For manuscripts utilizing custom algorithms or software that are central to the research but not yet described in published literature, software must be made available to editors and reviewers. We strongly encourage code deposition in a community repository (e.g. GitHub). See the Nature Research guidelines for submitting code & software for further information.

Data

Policy information about availability of data

All manuscripts must include a data availability statement. This statement should provide the following information, where applicable:

- Accession codes, unique identifiers, or web links for publicly available datasets
- A list of figures that have associated raw data
- A description of any restrictions on data availability

Additional data supporting the findings described in this manuscript are available in the Supplementary Information. All other data are available from the authors upon reasonable request. Source data are provided with this paper.

Field-specific reporting

Life sciences study design

All studies must disclose on these points even when the disclosure is negative.

Sample size	No statistical methods were used to predetermine sample size for in vitro and in vivo experiment. Sample sizes were chosen based on existing procedures and standards in the field.
Data exclusions	No data was exclusion.
Replication	ChIP assay and reporter assays were repeated at least three independent experiments. The qPCR analyses such as confirmation of knockout and overexpression, the expression change of known cartilage marker genes, and confirmation of miRNA expression of array results were performed in triplicate in a single experiment. The others were performed independently at least twice in duplicate or triplicate, as indicated in the figure legends. Other experiments were performed with at least three distinct samples. All attempts at replication were successful.
Randomization	Randomization was not required in this study, because we analyzed all experiments performed.
Blinding	Quantification of histological changes in the joint tissues was performed by two independent observers blinded to the experimental conditions. For histological analyses (supplementary figures 10, 12), sample collection and analysis were performed by different researchers without any description. For other studies, investigators were not blinded during the experiments. Blinding was not relevant for these studies, as no subjective rating of data was performed.

Reporting for specific materials, systems and methods

We require information from authors about some types of materials, experimental systems and methods used in many studies. Here, indicate whether each material, system or method listed is relevant to your study. If you are not sure if a list item applies to your research, read the appropriate section before selecting a response.

Materials & experimental systems

n/a	Involved in the study	
☐	☒	Antibodies
☐	☒	Eukaryotic cell lines
☒	☐	Palaeontology and archaeology
☐	☒	Animals and other organisms
☐	☒	Human research participants
☒	☐	Clinical data
☒	☐	Dual use research of concern

Methods

n/a	Involved in the study	
☒	☐	ChIP-seq
☒	☐	Flow cytometry
☒	☐	MRI-based neuroimaging

Antibodies

Antibodies used	ChIP: anti-SOX9 antibody (rabbit polyclonal, Millipore, ab5535, lot number 2654300), normal rabbit IgG (Wako, 148-09551), IHC: anti-HIF-2α antibody for mouse samples (rabbit polyclonal, Abcam, ab199, lot number GR267752-4), anti-HIF-2α antibody for human samples (rabbit polyclonal, Abcam, ab20654, lot number GR42186-1), anti-MMP13 antibody (rabbit polyclonal, Abcam, ab39012, lot number GR157514-7), Alexa Flour 488 conjugated donkey anti-rabbit antibody (Donkey polyclonal, Life Technologies, A21206, lot number 1723019).
Validation	anti-SOX9 antibody (ab5535): This is highly published antibody. This antibody is commonly used for ChIP and ChIP-seq in mouse chondrocytes (e.g. PMID: 26146088).<br>anti-HIF2A antibody (ab199): This antibody has been validated for IHC in lung of HIF2A KO mice (PMID: 27150457). This has also been validated for IHC in other murine tissues (e.g. PMID: 28813675, 26715642).<br>anti-HIF2A antibody (ab20654): This antibody has been validated for IHC in murine tissues (e.g. PMID: 28587437, 26846782).<br>anti-MMP13 antibody (ab39012): The antibody is commonly used for IHC in human tissues including OA cartilage (e.g. PMID: 28941045).

Eukaryotic cell lines

Policy information about cell lines

Cell line source(s)	HEK293FT (Thermo Fisher; R70007)
Authentication	The cell line was not authenticated.
Mycoplasma contamination	Not tested for mycoplasma contamination.
Commonly misidentified lines (See ICLAC register)	No commonly misidentified cell lines were used in this study.

Animals and other organisms

Policy information about studies involving animals; ARRIVE guidelines recommended for reporting animal research

Laboratory animals	Mouse strain: C57BL6, BDF1, miR-455 knockout mice.<br>Age: One cell stage, embryonic day 16.5, postnatal day 0~2, adult (4-week-old ~ 6-month-old)<br>Sex: male and female.
Wild animals	No wild animals were used in the study.
Field-collected samples	No field collected samples were used in the study.
Ethics oversight	All mice were freely allowed to access to food, water and activity. Mice were maintained under a 12 h dark-light cycle and constant temperature (20-26 ℃) and humidity maintenance (40-60 %).

Note that full information on the approval of the study protocol must also be provided in the manuscript.

Human research participants

Policy information about studies involving human research participants

Population characteristics	Human articular cartilage specimens were obtained from the knee joints of 10 normal donors (mean ± SD age 40.2 ± 11.0 years; female 4, male 6) and from 11 OA grade IV donors (71.7 ± 11.4 years; female 6, male 5) who recently deceased. Human MSCs used in this study were purchased from Lonza (PT-2501) or have been isolated from iliac crest bone marrow obtained from normal adult donors as previously reported (https://www.ncbi.nlm.nih.gov/pmc/articles/PMC2786215/).
Recruitment	Human cartilage samples were obtained at autopsy from individual within 48 hours post-mortem under approval by the Scripps Human Subjects Committee. The cartilage samples were selected only by OA grade. In diseases such as OA, which become more severe with age, it is difficult to exclude age from potential selection bias.
Ethics oversight	Human studies received ethical approval by the Scripps Human Subjects Committee at The Scripps Research Institute. Human tissues and cells were obtained with approval by the Scripps Human Subjects Committee at The Scripps Research Institute. All study donors provided written informed consent, according to the Declaration of Helsinki.

Note that full information on the approval of the study protocol must also be provided in the manuscript.
